# Implant removal rate and contributing factors following pancarpal arthrodesis in 42 dogs (52 cases): a multicentric retrospective study

**DOI:** 10.1186/s13028-025-00829-2

**Published:** 2025-09-25

**Authors:** Hélène Dosseray, Paolo Camilletti, Lou Shana Elbaz, Emilie Hanot, Guillaume Ragetly, Bertrand Pucheu, Laetitia Boland, Kévin Minier

**Affiliations:** 1 Centre Hospitalier Vétérinaire Anicura Nordvet, rue Delesalle 1, La madeleine, 59110 France; 2 Clinique Vétérinaire Evidensia Oncovet, Avenue Paul Langevin, Villeneuve d’Ascq, 59650 France; 3 Centre Hospitalier Vétérinaire Evidensia Fregis, Rue de Verdun 9, Gentilly, 94250 France

**Keywords:** Arthrodesis, Carpus, Dogs, Explantation, Implant removal

## Abstract

**Background:**

Despite advancements in pancarpal arthrodesis implants, the postoperative complication rate remains high, and implant removal is often required. This study assessed the implant removal rate following pancarpal arthrodesis and identified its associated factors. Case records of 52 pancarpal arthrodesis procedures performed on 42 dogs at three veterinary centres between 2017 and 2023 were reviewed. The collected data included signalment, medical history, surgical techniques, and postoperative follow-up, which were categorised into perioperative, short-term, mid-term, and long-term periods. Additionally, the timing and indications for implant removal were documented. Univariable logistic regression analysis was performed to analyse the data and identify factors associated with implant removal.

**Results:**

The implant removal rate was 36.5%. The presence of orthopaedic injuries in the contralateral limb was not associated with implant removal. The interval between diagnosis and pancarpal arthrodesis was significantly associated with implant removal (mean delay: 368.5 and 47.5 days for explantation and non-explantation cases, respectively). Carpal arthrodesis angle showed a statistically significant association with explantation (median angle: 8.58° and 11.73° for explantation and non-explantation cases, respectively). Perioperative and short-term surgical site infections, perioperative and short-term cultures and sensitivities, and the need for additional perioperative antibiotic therapy showed a statistically significant association with explantation.

**Conclusions:**

This study confirms the high implant removal rate following pancarpal arthrodesis. Although infection may contribute to this, prompt intervention and careful attention to the carpal arthrodesis angle intraoperatively may reduce this risk.

## Background

Pancarpal arthrodesis (PCA) is a salvage procedure that surgically fuses the antebrachiocarpal, middle carpal, smaller intercarpal, and carpometacarpal joints [1, 2]. PCA is indicated in various conditions, such as trauma (e.g., hyperextension injuries, ligament damage, and non-repairable distal radial or articular fractures), degenerative and immune-mediated arthropathies, congenital malformations, developmental abnormalities, and neurogenic disorders [2–4]. A fundamental principle of arthrodesis is the rigid stabilisation of the joints, typically achieved by internal fixation [1, 5, 6]. Various implants have been used for PCA, including dynamic compression plates, hybrid dynamic compression plates, hybrid locking plates, CastLess PCA plates, stepped hybrid plates, and custom plates [1, 4, 5, 7]. External fixation is also employed for performing PCA [8]. Despite the development of commercially available implants to reduce the risk of complications after PCA, the postoperative complication rate, excluding those related to external coaptation, can be as high as 34–41% [9]. The most common PCA complication is surgical site infection (SSI), reported in up to 31% of cases [6, 7, 9]. Other complications include arthrodesis failure, implant loosening or failure, metacarpal fractures, excessive nail wear, clicking, implant discomfort, soft tissue reaction over the plate, and persistent lameness [6, 9–14]. Implant removal (IR), also known as explantation, is commonly performed to address PCA-related complications. No previous studies have directly investigated the prevalence of PCA-IR and its associated factors. However, a survey on partial carpal arthrodesis investigating these parameters reported an IR rate of 41.2% [15]. Additionally, retrospective studies on PCA have indirectly suggested IR rates ranging from 12 to 33% [1, 4, 6, 7, 9, 10, 12, 16]. Extrapolating the IR rate from many studies is challenging, as it is often not reported as a major complication, and long-term follow-up data are frequently missing.

This study aimed to confirm the high, indirectly reported IR rate after PCA and to investigate its associated factors. We hypothesised that SSI and previous surgical carpal stabilisation are associated with the need for non-elective explantation.

## Methods

### Medical record search and inclusion criteria

The case records of dogs undergoing pancarpal arthrodesis at three veterinary centres from January 2017 to December 2023 were reviewed. Data were collected from hospital medical records and telephone interviews with the owner and/or referring veterinarian. Cases were excluded if they had a follow-up period of less than 3 months, underwent pancarpal arthrodesis with an external skeletal fixator, experienced perioperative death or euthanasia, or had a full ipsilateral thoracic limb amputation during the perioperative period. The collected data included signalment, dog activity (pet dog versus working dog), and previous carpal surgical stabilisation procedures, such as external fixation, ligament prosthesis, and PCA. Clinical examination findings included limb lateralisation, PCA aetiology (traumatic or non-traumatic), and the type of carpal instability, classified as dorsopalmar, mediolateral, or both. Other pathologies, including antebrachial growth deformity and carpal bone subluxation, were also recorded. Additionally, the presence of orthopaedic injuries in the contralateral thoracic limb was also recorded, including cases of amputation, bilateral hyperextension injury with or without PCA, antebrachial angular limb deformity, and polyarthritis. Additional preoperative diagnostic evaluations of the carpus, including biopsy and arthrocentesis, trauma-related carpal wound, and culture and sensitivity (C&S) testing, were documented. The age at diagnosis, date of PCA surgery, and the interval between diagnosis and surgery (calculated in days) were also recorded. Collected surgical data included perioperative antibiotic administration, plate position, plate type, use of autologous bone graft or biomaterials (bone substitute), PCA plate angle, carpal arthrodesis angle (CAA) [1], PCA metacarpal coverage (≤ 53% or > 53%), and the use of a sterile, iodine-impregnated adhesive surgical drape (3 M™ Ioban™ 2). Plate types were classified as traditional plates—such as dynamic compression plate, locking compression plate, and veterinary cuttable plate—or new-generation plates, which included all other plate types not previously mentioned. PCA surgeries were performed by an ECVS (European College of Veterinary Surgeons) diplomate, a supervised resident, or a surgeon holding a nationally recognized postgraduate qualification; the operating surgeon was recorded for each case and categorised anonymously as Surgeon 1 to Surgeon 6. Postoperative records included antibiotic and anti-inflammatory drug administration, external coaptation (such as soft padding, Robert Jones bandage, a splint, and an external fixator, along with any associated complications), and the duration of postoperative exercise restriction (≤ 3 months or > 3 months). Decisions regarding drug administration and external coaptation were determined at the surgeon’s discretion. The follow-up period was organised into four phases: the perioperative (PO) period (0–3 months), the short-term (ST) period (3–6 months), the mid-term (MT) period (6–12 months), and the long-term (LT) period (> 1 year) [17]. During each phase, data collection included the presence of a fistula or deep wound dehiscence, SSI based on standardised surveillance criteria from the Centres for Disease Control and Prevention’s National Nosocomial Infections Surveillance System [18], follow-up C&S sample and positivity, additional antibiotic therapy, lameness, implant-associated discomfort, and the requirement for total IR. The delay between surgery and IR, the indications for IR, and implant C&S positivity were also documented. Patients with partial IR were not classified as IR.

**Fig. 1:**
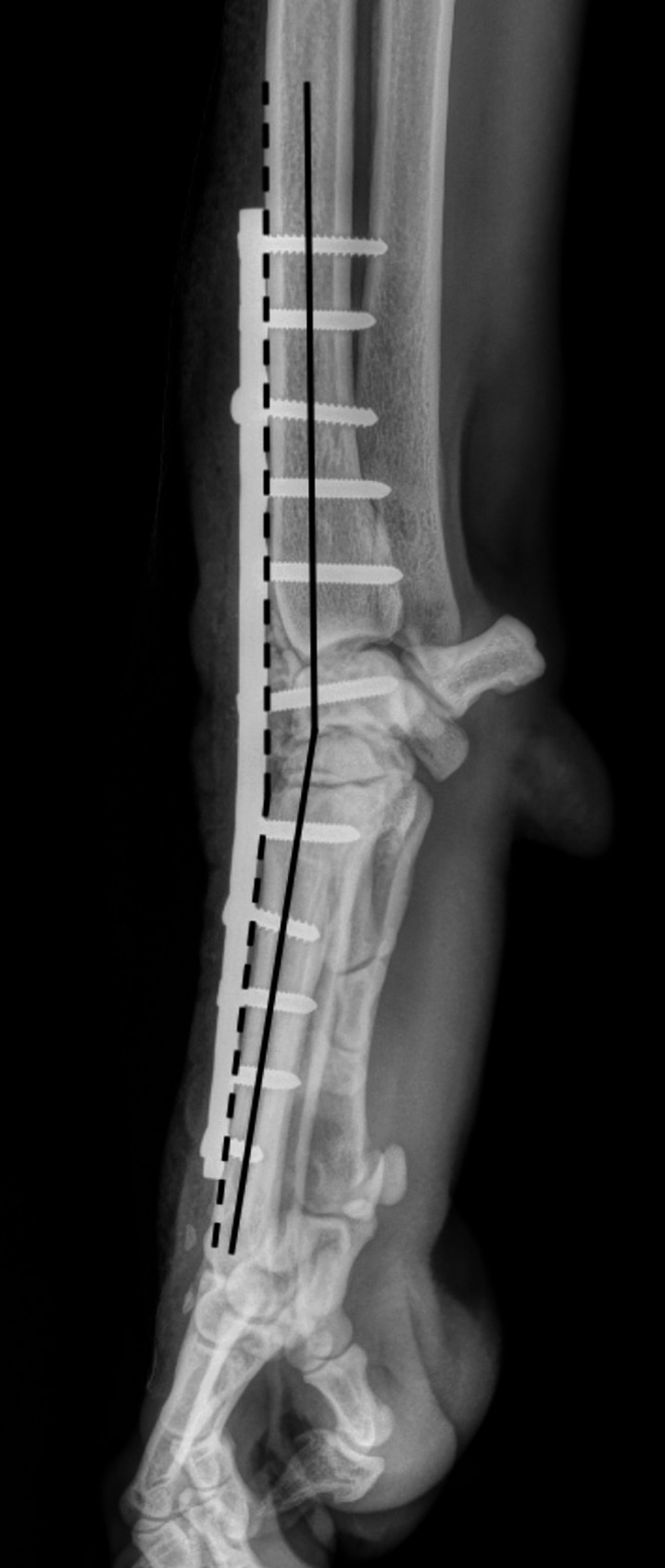
Measurement of PCA Plate Angle and Carpal Arthrodesis Angle (CAA) Radiographic illustration depicting reference lines for measuring the PCA plate angle and carpal arthrodesis angle (CAA). The PCA plate angle is the acute angle formed as a supplementary angle relative to these two dashed lines. In contrast, the CAA represents the acute angle of the supplementary angles associated with the two solid lines. The PCA plate angle was measured between the proximal and distal segments of the plate, while the CAA was determined using the anatomical axes of the distal radius and metacarpal bone in a mediolateral projection.

A diplomate of the European College of Veterinary Diagnostic Imaging conducted a blind review of each available radiograph, evaluating carpometacarpal, intercarpal, and antebrachial carpal bone fusion, as well as metacarpal bone fractures, implant failure, and radiographic signs of infection. Bone fusion was assessed by new bone bridging the joint space and classified as present or absent. Joint fusion was considered present if at least one bone per row was involved. Total fusion was defined as the fusion of all three joint spaces. Total fusion was considered absent if any joint space remained unfused. Radiographic signs of infection included (1) soft tissue swelling at the surgical site, observed in ST, MT, and LT periods (excluding the PO period), (2) osteolysis around the surgical implants, and (3) irregular periosteal new bone formation. Infection was deemed radiographically present if at least two of the three criteria were met. Implant failure was defined as an implant fracture and/or migration. The PCA plate angle, CAA, and metacarpal coverage were measured on immediate postoperative radiographs. The PCA plate angle was defined as the angle between the distal part of the plate along the metacarpal bone and the tangent of the proximal plate portion along the radius, reflecting the bending of the plate over the carpus on mediolateral projections. The CAA was measured from mediolateral projections using the anatomical axis of the distal radius and metacarpal bone [1] (Fig. 1). Metacarpal coverage of the surgical plate was calculated as the length of the plate covering the metacarpal bone divided by the total metacarpal bone length and categorised as either > 53% or ≤ 53%. PCA follow-up was considered complete under the following conditions: explantation, loss to follow-up, follow-up period extending beyond 31 December 2023, or if the patient died or was euthanised. The PCA follow-up duration was recorded in days.

### Data analysis

Analyses were performed using R software (version 4.2.1, R Core Team 2022). Although data were collected across four distinct follow-up periods, explantation was analysed as a single binary variable (0 = no explantation, 1 = explantation), indicating whether IR occurred at any point during the follow-up. Qualitative explanatory variables were categorised into two (such as antibiotic administration: Y/N) or three categories (such as instability status: mediolateral, dorsopalmar, or other). Data overdispersion was assessed before evaluating the significance of the variable effects using a threshold of two to detect overdispersion. To determine the significance of the explanatory variables at the 5% threshold (α), an univariable logistic regression model was constructed using the glm function [19]. Odds Ratios (ORs) were reported for variables significantly associated with explantation (*P*-value < 0.05).

## Results

### Population

Fifty-two PCAs (out of sixty-five) on 42 dogs were included in the study. Table 1 presents the total number of cases monitored during each period, with 18 cases not reaching the LT period (> 12 months). For non-explantation cases, follow-up data for the ST, MT, and LT periods were unavailable for two cases, and LT follow-up data were unavailable for four. The mean PCA follow-up duration was 369 days (standard deviation [SD]: ±400) for explantation cases and 670 days (SD: ±505) for non-explantation cases. The most common breeds were Belgian Shepherds (*n* = 6), Australian Shepherds (*n* = 4), and Border Collies (*n* = 4). Twenty other breeds were also included in the study. The study population consisted of 27 males and 15 females. The total mean weight was 26.2 kg (SD: ±11.6). The mean weight for explantation and non-explantation patients was 24.9 kg (SD: ±11.6) and 27 kg (SD: ±11.7), respectively. The mean age at diagnosis and at the time of PCA surgery was as follows: Explantation cases: 5.37 years at diagnosis and 6.38 years at surgery. Non-explantation cases: 5.7 years at diagnosis and 5.79 years at surgery. There were 4 working dogs and 32 pet dogs in the study. No significant associations were noted between the explantation status and breed, sex, weight, age at diagnosis, age at PCA, or dog activity.

**Table 1:** Distribution of explantation and non-explantation cases across defined follow-up periods, along with explantation rates

Follow-up period	Explantation cases (*n*)	Non-explantation cases (*n*)	Total cases for each period (*n*)	Explantation rate (%)
Perioperative (PO)	1	51	52	1.9% (1/52)
Short-term (ST)	6	43	49	12.2% (6/49)
Mid-term (MT)	5	38	43	11.6% (5/43)
Long-term (LT)	7	27	34	20.6% (7/34)
Total (PO/ST/MT/LT)	19	33	52	36.5% (19/52)

Abbreviations: n, number

Legend: percentages represent the proportion of explantation cases within each time period. Differences in total case numbers across periods are due to varying follow-up durations and the exclusion of cases explanted before reaching later time points

### Distribution and causes of IR

Nineteen explantations (36.5%) were recorded among the 52 cases. Table 1 describes the explantation rate across each period and indicates a higher occurrence during the MT and LT periods (12 out of 19 cases) than during the PO and ST periods (seven cases). All cases of IR, except one, resulted from the presence of SSI accompanied by implant-associated discomfort, lameness, or implant/screw loosening; the remaining case was attributed solely to lameness and plate discomfort. Among the explantation cases with recorded C&S results, nine had (47%) positive C&S results, with *Staphylococcus pseudintermedius* (*S. pseudintermedius*) being identified in six of those cases.

### Pre-surgical contributing factors

Among the 52 cases, 10 (19%) had previously undergone carpal stabilisation, such as external fixation, ligament prosthesis placement, or prior ipsilateral PCA; however, no significant association was observed between this factor and IR. In these instances, PCA was deemed necessary either due to failure of the implant or prosthesis or as a subsequent procedure following the removal of the external fixator. Limb lateralisation, defined as PCA involvement of either the right or left thoracic limb, was not associated with explantation. Furthermore, orthopaedic injuries sustained in the thoracic limb opposite to the PCA site were not linked to the need for explantation. In total, 22 cases (42%) involved the contralateral thoracic limb. Nine patients underwent bilateral PCA, accounting for 18 cases; of these, eight had staged surgeries, while one underwent a single-session bilateral PCA. Among the remaining four cases with contralateral thoracic limb involvement, conditions included forelimb amputation, polyarthritis, absence of PCA treatment, and antebrachial angular deformity. Of the 50 cases with a known aetiology, 41 cases (82%) were classified as traumatic, with falls from height being the most frequently reported cause (24 cases). Among these, six involved bilateral carpal injuries. Other traumatic causes included running or hunting injuries, bite or wire wounds, distal radial fractures, and road-traffic accidents. Among non-traumatic cases (18%), arthropathy—either erosive, degenerative, or associated with polyarthritis—was the most common cause, followed by antebrachial growth deformity. No significant difference in IR rates was observed based on the aetiology of PCA, whether traumatic or non-traumatic. Carpal conditions leading to PCA were identified in 50 patients and categorised as dorsopalmar instability, mediolateral instability, combined dorsopalmar and mediolateral instability, or other pathologies. Dorsopalmar instability, mediolateral instability, dorsopalmar and mediolateral instability, and other pathologies were diagnosed in 23 (46%), 8 (16%), 15 (30%), and 4 cases (8%), respectively. The instability status was not significantly associated with explantation. Additional preoperative diagnostic evaluations of the carpus (biopsy or arthrocentesis) were conducted in six cases (11%); trauma-related carpal wounds were documented in 11 cases (21%); and C&S testing was performed in five cases (9.6%), with one case yielding a positive result. No significant associations were found between IR and the following factors: additional preoperative diagnostic evaluations of the carpus, trauma-related carpal wounds, or preoperative C&S samples. However, the time to surgical intervention was significantly associated with IR (*P* = 0.04, OR: 1.01, 95% CI: [1.00, 1.02]), with a mean delay of 368.5 days (SD: ±619.7) for explantation cases and 47.5 days (SD: ±64.7) for non-explantation cases.

### Surgical contributing factors

All cases received amoxicillin–clavulanic acid or cefalexin every 90 min from induction to wound closure, then 8 hourly for 24 h, followed by oral administration. The plate was positioned dorsally in all cases except one. PCA metacarpal coverage exceeded 53% in all but two cases. No statistical association was observed between these two factors. Traditional plates were utilised in only 4 of the 52 cases, whereas new-generation plates (hybrid plates or CastLess PCA plates) were employed for the remaining cases. In nine cases (17%), iodine-impregnated adhesive surgical drapes (3M™ Ioban™ 2) were applied, all at the same centre. Ipsilateral humeral bone grafts or bone substitutes (Biocera-vet^®^ bone surgery Ready-to-Use [RTU], TheraVet, Gosselies, Belgium) were documented in 46 surgeries, with 19 cases out of the 46 (41%) receiving bone substitutes and 27 (59%) receiving bone grafts. Notably, none of these three factors—plate type, use of adhesive surgical drape, and graft type—were associated with IR. This study found that the median PCA plate angle for explantation cases was 3.6° (SD: ±4.2) and 6.6° (SD: ±3.1) for non-explantation cases. Statistical analysis revealed a significant association between the PCA plate angle and explantation cases (*P* = 0.01, OR: 0.79, 95% CI: [0.65,0.93]). Additionally, the median CAA was 11.73° (SD: ±3.78) for non-explantation cases and 8.58° (SD: ±4.86) for explantation cases, showing a significant association with explantation status (*P* = 0.02, OR: 0.7891, 95% CI: [0.61,0.94]). Table 2 shows the total number of PCA procedures performed by each operating surgeon and the number of explantations for each surgeon. This information is provided descriptively, as surgeon was not included as an explanatory variable in the regression analysis due to the limited sample size.

**Table 2:** Number of PCA procedures performed and explantations recorded per surgeon

Surgeon	PCA procedures (*n*)	Explantations (*n*)
Surgeon 1	10	2
Surgeon 2	6	6
Surgeon 3	2	0
Surgeon 4	30	11
Surgeon 5	3	0
Surgeon 6	1	0

Abbreviations: PCA, pancarpal arthrodesis; n, number

Legend: Total number of PCA procedures performed by each surgeon and the corresponding number of cases in which explantation was required

### Radiographic factors

Fusion of the carpometacarpal and intercarpal joints was achieved between 9 and 12 weeks in 39 of 42 cases, while fusion of the antebrachiocarpal joints occurred between 17 and 30 weeks in 37 of 38 cases. Total fusion was achieved at 30 weeks in all 38 cases recorded, with no significant association between fusion and IR. Radiographic signs of PO and ST infections were observed in 22 and 14 cases, respectively. PO infection radiographic signs were associated with IR (*P* = 0.022, OR:5.64, 95% CI: [1.40,29.50]), whereas ST infection radiographic signs were not associated with IR (*P* = 0.059, OR:5.98, 95% CI: [1.06, 50.03]).

### PO & ST contributing factors

Table 3 summarises the descriptive statistics for the explantation and non-explantation case groups, as well as the significance of each qualitative PO and ST factor associated with explantation. All cases received PO non-steroidal anti-inflammatory drugs for 5–20 days. All but three cases received PO antibiotic therapy for 7 days to 1 month, depending on the surgeon’s preferences and pre-surgical C&S results. After completion of PO antibiotic therapy and in cases of suspected SSI during the PO, ST, MT, and LT periods, additional antibiotic therapy was administered based on positive C&S results, instances where C&S was declined for financial reasons, and cases of highly suspected culture-negative infection. These additional antibiotic therapies included fluoroquinolones (marbofloxacin, enrofloxacin), tetracyclines (doxycycline), β-lactams (amoxicillin-clavulanic acid, cephalexin), aminoglycosides (gentamicin), or lincosamides (clindamycin). PO SSI was observed in 26 cases (50%) and was associated with explantation (*P* = 0.015, OR: 4.67, 95% CI: [1.41, 17.59]). PO C&S was performed in 9 of 51 cases (17%), with positive results in 5 cases. Additional antibiotic therapy was administered in 13 of the 51 cases (25%). Both postoperative C&S and additional antibiotic therapy were significantly associated with IR (*P* = 0.005 and 0.009; OR: 22.2 and 6.3; 95% CI: [3.57,443] and [1.67, 27.81], respectively). Among ST factors, fistula formation or deep wound dehiscence was documented in 14 of 47 cases, while ST SSI was observed in 19 of 47 cases. ST C&S was performed in 6 of 47 cases, with positive results in 3 cases. ST fistula formation (*P* = 0.001; OR, 11.24; 95% CI, [1.67;27.81]), ST SSI (*P* = 0.006; OR, 6.33; 95% CI [2.81;54.24]), and C&S sample (*P* = 0.02; OR, 13.60; 95% CI [1.06;25.87]) were all significantly associated with IR.

**Table 3:** Descriptive statistics for explantation and non-explantation groups, assessing qualitative PO and ST factors’ significance

Table 3		Non-explantation cases	Explantation cases	*P* values
PO factors	Postoperative external coaptation (N/Y)	7/26	6/13	0.41
	Complications related to external coaptation (N/Y) †	18/8	9/4	0.99
	Postoperative rest period (< 3months/>3months)	30/3	17/2	0.87
	Postoperative Antibiotic therapy (Amoxicillin-Clavulanic Acid/Cefalexin/Doxycycline/ Marbofloxacine) †	9/20/1/0	10/7/1/1	0.55
	PCA wound complication (N/Y)	26/7	11/8	0.11
	Wound graft complication (N/Y) †	17/1	14/2	0.49
	Fistula (N/Y) †	25/7	9/10	0.84
	Infection radiographic signs (N/Y) †	17/11	3/11	0.022 (OR: 5.64) *
	SSI (N/Y) †	20/12	5/14	0.015 (OR: 4.67) *
	C&S sample (N/Y) †	31/1	11/8	0.005 (OR: 22.20) *
	Positive C&S (N/Y) †	31/1	15/4	0.069
	Implant failure (N/Y) †	25/3	11/3	0.36
	Metacarpal fracture (N/Y) †	27/1	11/3	0.10
	Additional Antibiotic therapy (N/Y) †	28/4	10/9	0.009 (OR: 6.3) *
	Plate discomfort (N/Y) †	28/4	14/5	0.22
	Lameness (N/Y)	12/21	7/12	0.97
ST factors	Fistula (N/Y) †	27/4	6/10	0.001 (OR: 11.24) *
	Infection radiographic signs (N/Y) †	9/6	2/8	0.059
	SSI (N/Y) †	23/8	5/11	0.0065 (OR: 6.33) *
	C&S sample (N/Y) †	30/1	11/5	0.02 (OR: 13.60) *
	Positive C&S (N/Y) †	29/1	14/2	0.26
	Implant failure (N/Y) †	12/3	5/3	0.37
	Metacarpal fracture (N/Y) †	15/0	8/2	0.99
	Additional Antibiotic therapy (N/Y) †	26/5	11/5	0.24
	Plate discomfort (N/Y) †	24/7	12/4	0.85
	Lameness (N/Y) †	18/13	7/9	0.35

Abbreviations: PO, perioperative; ST, short-term; SSI, surgical site infection; C&S, culture and sensitivity; N, no; Y, yes; OR, odds ratio

†: Due to missing data on this factor, the total sample size was less than 52 cases

*: *P* value < 0.05

Legend: Each cell in the explantation and non-explantation columns present the number of dogs classified as “No/Yes” (N/Y) or categorical values (type of antibiotic or postoperative rest period) for the corresponding qualitative factor. Due to missing data, totals may not equal 52 for all factors. The “*P*-value” column reports the result of a univariable logistic regression analysis assessing the association between each factor and implant removal. Where significant (*P* < 0.05), odds ratios (ORs) with 95% confidence intervals (CIs) are provided in parentheses

### MT and LT factors

Table 4 summarises the descriptive analyses of the MT and LT periods.

**Table 4:** Descriptive analysis of MT and LT factors for the explantation and non-explantation case groups

Table 4		No-explantation cases	Explantation cases
MT factors	Lameness (N/Y)	21/10	4/6
	Fistula (N/Y)	30/1	4/6
	SSI (N/Y)	27/4	3/8
	C&S sample (N/Y)	31/0	9/1
	Positive C&S (N/Y)	31/0	10/0
	Additional antibiotic therapy (N/Y)	30/1	8/2
	Plate discomfort (N/Y)	24/7	6/4
	Infection radiographic sign (N/Y)	0/4	0/6
	Implant failure (N/Y)	2/2	2/4
	Metacarpal fracture (N/Y)	4/0	5/1
LT factors	Lameness (N/Y)	19/8	1/5
	Fistula (N/Y)	25/2	0/7
	SSI (N/Y)	22/5	0/7
	C&S sample (N/Y)	25/1	7/0
	Positive C&S (N/Y)	26/0	7/0
	Additional antibiotic therapy (N/Y)	27/0	5/2
	Plate discomfort (N/Y)	20/7	3/4
	Infection radiographic sign (N/Y)	3/4	0/5
	Implant failure (N/Y)	3/4	1/4
	Metacarpal fracture (N/Y)	7/0	5/0

Abbreviations: MT, mid-term period; LT, long-term period; SSI, surgical site infection; C&S, culture and sensitivity; N, no; Y, yes

Legend: Values are displayed as “No/Yes” (N/Y), indicating the number of cases with and without the recorded factor. Missing data may result in totals differing from group sizes

### Statistical analysis

No evidence of over-dispersion was observed in the significance tests. However, due to the limited number of cases, incorporating multiple explanatory variables into the model was statistically unfeasible. Spearman’s correlation analysis was performed for all factors to assess the potential confounding effects. Among these, strong positive correlations (with coefficients ranging from 0.8 to 1) were observed between ST SSI and ST infectious radiographic signs, PO SSI and PO infectious radiographic signs, and the ages at trauma and surgery. Each of these is logically associated with the others. Additionally, the remaining sample size was inadequate for statistical analysis of the association between MT and LT period factors and explantation.

## Discussion

This study identified an IR rate of 36.5% after PCA. Significant factors associated with IR included a lower PCA plate angle and reduced CAA. A longer interval between diagnosis and surgical intervention was also a contributing factor. Additionally, PO and ST SSI, PO infection radiographic signs, PO and ST C&S, and the use of additional PO antibiotic therapy were all linked to IR. Although prior surgical carpal stabilisation was not statistically significant, a notable association was observed.

In veterinary medicine, the criteria for IR remain poorly defined. Current human medical guidelines recommend against the routine removal of orthopaedic implants and recommend explantation only under specific circumstances, such as implant-associated discomfort, implant failure, metal allergies, functional limitations, or infection [20–22]. In small animal practice, IR is mainly performed in cases of complications, such as implant loosening/failure, interference with bone growth, soft tissue irritation, or infection [23]. However, formal guidelines for IR have yet to be established [24]. As a result, the decision to remove an implant is left to the surgeon’s discretion, potentially impacting the prevalence of explantation procedures. In this study, all surgeries were conducted by skilled orthopaedic surgeons, minimizing variability related to surgeon expertise. With infection being the main reason for IR, the decision-making process was mostly consistent, with minimal impact from individual surgeon preferences.

IR rates for common procedures such as Tibial Plateau Levelling Osteotomy (TPLO) (2.7–7.4%) [25–27] and tibial tuberosity advancement (1.8%) [28] were lower than those for the procedure examined in this study, which had an IR rate of 36.5%. This finding is consistent with previously reported PCA explantation rates [1, 4, 6, 7, 9, 10, 12, 16, 29]. In summary, previous PCA studies indirectly reported explantation rates, as they were not the primary focus of these studies, ranging from 12.4 to 33%, with case numbers varying from 11 to 219 and a median follow-up of up to 49 months [1, 4, 6, 7, 9, 10, 12, 16, 29]. This study differs by including a larger sample size or a longer median follow-up, which may explain the higher explantation rates observed. Of note, the high standard deviation in follow-up time observed in our cohort was primarily due to follow-up being truncated at the time of IR. Consequently, cases requiring early explantation inherently had shorter follow-up durations. Furthermore, since this is a retrospective study, the follow-up periods were not standardized and depended on the availability of records. The PCA explantation rate was also comparable to the 21.25% rate reported for open fractures stabilised with internal fixation [24], suggesting that contamination or infection may be key contributing factors, as confirmed in this study.

Most PO and ST infection-related factors, including SSI, radiographic signs of infection, use of additional antibiotic therapy, and C&S samples, were significantly associated with explantation. This finding aligns with that of a previous study on IR and associated risk factors following open fracture fixation, which reported a relative risk of 2.77 (95% CI: 1.25;6.15) for explantation in dogs with postoperative infections [24]. Additionally, research on microbial colonisation following osteosynthesis in small animals found that nearly half of the implants exhibited significant microbial contamination [23]. The same study also revealed that the location and type of plate influenced the likelihood of infection development [23]. Infections were more common in forelimb implants than those in hindlimb implants, and structures distal to the elbow were more frequently affected than those proximal to it [23]. Similarly, a large study comparing hybrid compression and castless plates for PCA [9] identified SSI as the most common major postoperative complication, occurring in 17.6% and 19% of cases, respectively. Additionally, another study on PCA in working dogs reported SSI as the only postoperative complication [29]. This is consistent with the high rate of PCA explantation in this study and its associated causes. In this study, SSI was identified as the reason for explantation in all but one case. SSI classification in this study followed the standardised method from the Centers for Disease Control and Prevention’s National Nosocomial Infections Surveillance System [18], which allows diagnosis based on clinical signs without requiring a positive culture. This may explain why only 47% of IRs had a positive culture result despite clinical evidence consistent with infection. Additionally, *S. pseudintermedius* was identified in six of nine culture-positive explantation cases. These findings are consistent with a previous report identifying *S. pseudintermedius* as the most predominant pathogen in SSIs following orthopaedic procedures in dogs and cats [30]. A postoperative infection diagnosis significantly increases the likelihood of requiring explantation, highlighting the importance of focusing on infection prevention measures during case management [24]. Anatomical and surgical factors may contribute to the increased susceptibility of the carpal region to infections. The thin, soft tissue layer in this area reduces vascularity, potentially hindering healing and making the tissue more susceptible to contamination and infection [9]. In cases of mild incisional infections following PCA surgery, there is an increased risk of bacterial contamination of the bone plate, which may promote biofilm formation. This can impede the effective resolution of what would otherwise be a self-limiting infection [26]. Additionally, PCA requires cartilage surface burring and bone grafting procedures, which take significantly longer than other common orthopaedic procedures and carry an increased risk of bacterial exposure [9, 26, 31]. Thermal bone necrosis induced by cartilage burring increases the risk of infection [26].

ST infectious radiographic signs were not associated with IR; however, a trend was observed (*P* = 0.059). This observation could be attributed to the smaller sample size during the ST period, likely reducing the statistical power to detect a significant association. PO fistulas were not associated with explantation, which may be related to ambiguous reporting of fistulas, complicated and delayed wound healing in the PO period, or possibly type II error. C&S samples during the PO and ST periods—both associated with IR—were not collected for all cases of suspected SSI. This may have introduced selection bias, as more severe or clinically apparent cases were more likely to be sampled. Additionally, positive C&S results, rather than merely submitting C&S samples, were not associated with IR, potentially due to factors such as prior antibiotic treatment or culture-negative infections. In human medicine, culture-negative periprosthetic joint infections have a prevalence of 27–55% [32]. The use of ST additional antibiotic therapy was not associated with IR. Once fusion is achieved, surgeons may prefer explantation over medical management of the SSI. This preference could explain the higher number of explantation cases observed during the MT and LT periods. In the non-explantation group, MT and LT infection-related factors were identified. This may indicate an owner’s reluctance to proceed with PCA plate removal, even with clinical signs suggestive of infection, reflecting a tendency to delay or decline further intervention despite potential complications. This hesitation could stem from inadequate patient management or financial considerations. In the context of PCA, additional indications for explantation include soft tissue irritation, implant loosening, sepsis, and persistent lameness [10, 11, 33]. Lameness was reported in both explantation and non-explantation groups during the MT and LT periods. This is a documented complication that may or may not lead to explantation, as it can be mechanical in nature after PCA [6, 9]. The PCA angle, particularly the steeper angles, may influence this phenomenon; therefore, further research should explore this aspect.

Although the findings approached statistical significance (*P* = 0.09), this study did not establish a significant association between previous surgical carpal stabilisation and IR. This result may be explained by the limited number of cases with prior stabilisation (*n* = 10), which likely reduced the statistical power to detect a meaningful association. In a previous study, 66.7% of removed implants were infected after repeated osteosynthesis procedures [23]. The elevated infection rate likely resulted from compromised local soft tissue conditions due to previous surgical interventions. Poorly vascularized scar tissue may increase infection risk, particularly with metallic implants, by impairing immune defence and limiting antibiotic penetration [23]. This raises the question of whether intraoperative C&S should be routinely performed in such cases. More broadly, its relevance in first-intention PCA procedures warrants further investigation, particularly in patients with impaired soft tissue quality.

In this cohort, both the PCA plate angle and CAA influenced IR, with a higher explantation rate in patients presenting with a lower degree of hyperextension. According to the general rule that the post-PCA hyperextension angle should approximate the physiological angle of 10–15 degrees [10, 34, 35], deviations from this value may lead to suboptimal outcomes. The findings of this study reinforce this principle. A decrease in physiological carpal extension may cause excessive stress on the metacarpophalangeal joints, resulting in poorer outcomes and prompting surgeons to remove the implant [36]. Conversely, a greater degree of extension increases the moment arm of forces relative to the plate bend [37]. Reducing the hyperextension angle can reduce the stress exerted at the distal end of the plate, thereby lowering the loading moment at this critical point [38]. This adjustment may help mitigate the risk of complications, such as implant failure [38]. Additionally, since the CAA was measured only in the immediate postoperative period, it would have been valuable to assess it at later time points, as it may have increased slightly over time. The PCA plate angle depends on the plate design and plate contouring. Whether a PCA plate should be contoured remains a subject of debate [1]. Due to the design of the tapered distal plate, a hybrid plate generally does not require contouring, as it naturally creates an angle between 5° and 10° [1, 5, 33]. However, surgeons may elect to contour the plate as needed to achieve an optimal CAA.

A delay between diagnosis and surgical intervention was associated with explantation. Specifically, the longer the delay, the higher the possibility of explantation. Although the underlying pathology was not a significant factor in this study, the aetiology of PCA and the time from diagnosis to surgery may be linked, as treatment timelines differ between polyarthritis and trauma management. In human medicine, inflammatory arthritis, particularly polyarticular disease, is associated with poor patient-reported outcomes [39]. Additionally, wrist arthrodesis with a dorsally applied plate in patients with inflammatory diseases is considered risky because of the delicate skin and the higher risk of infection. Conversely, a previous study found that a longer delay between diagnosis or injury occurrence and surgery did not significantly increase the risk of infection [23]. However, in their study, fracture was the main indication for surgical intervention, followed by arthrodesis. Arthrodesis is generally considered a salvage procedure and may only be pursued after a prolonged delay.

The findings of this study suggest a possible surgeon effect. Specifically, surgeon 6 had a 100% IR rate. Although the sample size was small (*n* = 6), two factors may have contributed to this outcome: (1) hesitancy to perform the procedure, resulting in delayed intervention, and (2) reduced PCA plate angles (and, consequently, reduced or absent plate contouring). In the present study, both surgical delay and a lower PCA plate angle were significantly associated with IR. These observations indicate that inter-surgeon variability in clinical decision-making and surgical technique, potentially influenced by differences in training and experience, may substantially affect postoperative outcomes.

This study indicated no significant impact of contralateral limb involvement on explantation. This variable has not been extensively investigated as a potential factor related to PCA explantation. Plate breakage has been reported in cases of bilateral PCA (single session and staged surgeries) [9]. In our study, plate breakage was observed in a single case, which also involved a bilateral PCA (staged surgery in this case). To investigate this issue further, future studies should specifically evaluate the outcomes and complications associated with single-session or staged bilateral PCA, as this may impact prognosis.

One of the main limitations of this study is its retrospective design, which restricts control over variables and may introduce selection or information bias. Furthermore, the lack of standardised treatment protocols, such as variations in implant types, antibiotic treatments, and postoperative care, across centres and surgeons may have influenced the observed outcomes. In this context, the heterogeneity in the type and duration of postoperative or additional antibiotic therapy prevented meaningful statistical analysis, and these variables were therefore recorded as a binary outcome (administered: yes/no). Future prospective studies with standardised approaches would help validate and expand upon these results. The relatively small sample size reduces the statistical power and the ability to detect true differences between groups, potentially leading to false-negative results (type II error). Furthermore, due to the limited sample size, it was not feasible to include the surgeon as an explanatory variable in the regression model without risking model instability and non-convergence; however, the explantation rates are provided per surgeon, and we acknowledge that investigating surgeon-specific effects would be valuable and should be addressed in future studies. Additionally, explantations occurring across four distinct periods were combined into a single binary response variable. Although this approach simplified the analysis, it may have introduced bias by not accounting for the specific associations of each factor with the corresponding period-specific explantation rates. A more detailed analysis that preserves the temporal dimension could provide greater insight into the relationship between each factor and explantation rates over time.

## Conclusions

Explantation following PCA is more prevalent than those associated with other orthopaedic procedures, mainly because of infection-related factors. Prompt surgical intervention and careful consideration of the angles of the PCA plate and carpal arthrodesis during surgery may help mitigate the risk of explantation and improve patient outcomes.

## Data Availability

The datasets used and/or analysed during the current study are available from the corresponding author on reasonable request.

## Declarations

### Prior publication

Data included in this article were previously presented and published in the Proceedings of the European College of Veterinary Surgeons Congress, Antwerp, Belgium, 3–5 July 2025.
